# Structure Differences of Water Soluble Polysaccharides in *Astragalus membranaceus* Induced by Origin and Their Bioactivity

**DOI:** 10.3390/foods10081755

**Published:** 2021-07-29

**Authors:** Zhili Sheng, Junmei Liu, Bao Yang

**Affiliations:** 1 College of Food Science and Engineering, Jilin Agricultural University, Changchun 130118, China; shengzl@scbg.ac.cn; 2 Guangdong Provincial Key Laboratory of Applied Botany, South China Botanical Garden, Chinese Academy of Sciences, Guangzhou 510650, China

**Keywords:** glycosidic linkage, monosaccharide composition, NMR, pectin

## Abstract

*Astragalus membranaceus* is a functional food with multiple bioactivities. It presents differentiated health benefits due to origins. Polysaccharides (APS) are the leading bioactive macromolecules of *A. membranaceus*, which are highly related to its health benefits. However, the effect of origin on the structural characteristics of APSs remains unclear. In this work, polysaccharides from four origins were isolated and identified by NMR. The results showed APSs of four origins had identical monosaccharide composition and glycosidic linkage. Rhamnogalacturonan II pectins and α-(1→4)-glucan were the dominant polysaccharides. However, the level of methyl ester in pectins varied to a large extent. The molecular weight profiles of APSs were also different. Inner Mongolia APS had the largest percentage of 20–40 kDa polysaccharides. Molecular weight and methyl ester level were two important parameters determining the difference of APSs from four origins. These results were helpful to recognize the origin-related quality of *A. membranaceus*.

## 1. Introduction

*Astragalus membranaceus* rhizome is widely recognized as a functional food and traditional Chinese medicine due to multiple health benefits. It has shown impressive biological activities and has been used for immunomodulation and cancer prevention [1]. *Astragalus membranaceus* is capable of exerting immune activity through restoring the impaired T cell functions in cancer patients [2]. The water-soluble polysaccharides (APS) can enhance the innate immune response by increasing the receptor expression [3]. This is an important therapeutic approach against cancer. *A. membranaceus* also performs well in anti-inflammatory, antioxidant, and antiviral effects [4]. *Astragalus* polysaccharides can regulate the signal transduction of anti-inflammatory and pro-inflammatory factors to interfere with various inflammatory diseases and can affect several pathway mediators of inflammation [5]. Due to their natural origins, an increasing number of studies have paid attention to the representative bioactive compounds, APS [6].

Polysaccharides are usually the main components of the plant cell wall. They construct dense physical structures against biotic and abiotic attacks. Furthermore, they play an important role in maintaining the human body in healthy status. Multiple functions have been reported for bioactive polysaccharides, such as antitumor, immunomodulation, antioxidation, and antiinflammation [7,8,9]. The structure features of polysaccharides are directly related to the health benefits. These structure features include the monosaccharide composition, glycosidic linkage, molecular weight, repeating unit, and branch chain [10]. For instance, glucose and fucose are two important residues in the side chain that bring Ganoderma lucidum polysaccharides immunomodulatory activity [11]. Mannose-rich exopolysaccharides from *Tremella* and rhamnose-rich exopolysaccharides from *Lactobacillus rhamnosus* can stimulate the immune system through receptors located on the macrophage [12]. The polysaccharides containing a high level of uronic acid usually display good antioxidant activity in vivo [13]. Several studies have documented that low molecular-weight polysaccharides show effective antioxidant activities [14,15].

In China, *A. membranaceus* mainly grows in north China [16]. The health benefits of *A. membranaceus* are closely related to ecological factors, including climate and the growing environment [17]. It has been reported that *A. membranaceus* from different origins show chemical and genetic diversity, which genetically differentiate their quality-related traits [18]. As polysaccharides are the leading biological macromolecules in *A. membranaceus*, their structural characteristics are responsible for the health benefits of this plant. However, relevant information in this regard is limited. Therefore, in this work, samples of *A. membranaceus* of four representative origins were collected. The water-soluble polysaccharides were extracted. The molecular weight and structural characteristics were analyzed by gel permeation chromatography and nuclear magnetic resonance spectroscopy (NMR). These results could explain how the structure and composition of polysaccharides changed due to their origin.

## 2. Materials and Methods

### 2.1. Extraction of Water-Soluble Polysaccharides 

The dried roots of *A. membranaceus* were collected from four origins (Shanxi, Inner Mongolia, Gansu, and Heilongjiang provinces). They situated between latitudes 37°24′–53°23′ N (Inner Mongolia), 32°31′–42°57′ N (Gansu), 37°27′–53°25′ N (Shanxi), 43°26′–53°33′ N (Heilongjiang) and longitudes 97°12′–126°04′ E, 92°13′–108°46′ E, 111°30′–113°09′ E and 121°11′–135°05′ E, respectively. The climates of Shanxi and Heilongjiang were characterized by humid regions with fertile soils, while Inner Mongolia and Gansu were characterized as arid and semi-arid climates. All the materials were evaluated according to the shape, diameter, and length. The reagents used in this study were analytically pure. The ethanol and trifluoroacetic acid were purchased from Macklin Biochemical Co., Ltd. (Shanghai, China). Phenol, m-hydroxyl diphenyl, pyridine, monosaccharide standards, hydroxylamine hydrochloride, acetic anhydride were purchased from Aladdin Reagent Inc. (Shanghai, China). Phosphate-buffered saline (PBS) was purchased from Gibco Life Technologies (Grand Island, NY, USA). Fluorescein sodium, Trolox, 1,1-diphenyl-2-picryldydrazyl (DPPH), L-glutamic acid, and 2,2′-azobis-amidinopropane (ABAP) were purchased from Sigma Chemical Co. (St. Louis, MO, USA).

The extraction of water-soluble polysaccharides was conducted according to the protocol of Yang et al. [19]. The dried roots of A. membranaceus (100 g) were crushed and subjected to extraction with 2 L of deionized water at 60 °C for 2 h. After filtration and centrifugation at 9000× *g* for 20 min, the supernatants were collected and concentrated at 60 °C by a vacuum rotary evaporator (Eyela N-1100 V-W, Tokyo Rikakikai Co. Ltd., Tokyo, Japan). Anhydrate ethanol was added to a final concentration of 60% (*v*/*v*) and incubated for 12 h at 4 °C in a refrigerator. The pellets were collected by centrifugation at 6000× *g* for 20 min, and freeze-dried to obtain the water-soluble polysaccharides (APS).

### 2.2. Chrominance Analysis

The color property was detected according to the protocol of Chen et al. with some modifications [20]. The L *, a *, and b * table color system was determined by using a colorimeter (Model CR-300; Minolta, Tokyo, Japan) and the standard of white plate was Y = 92.30, x = 0.3162, and y = 0.3323. The color difference (ΔE) between the Inner Mongolia sample and other origins was calculated with the standard L*, a*, b* values. The formula was ΔE = [ (ΔL*)2+ (Δa*)2+ (Δb*)2]^1/2^. For each treatment, polysaccharides were randomly chosen and the surface color was measured in triplicate. 

### 2.3. Determination of Polysaccharide Content

The polysaccharide content was determined by phenol–sulfuric acid protocol with glucose as standard [21]. Polysaccharide samples (0.2 mg) were dissolved in distilled water (0.2 mL), then 0.2 mL of 5% phenol solution was added and kept at room temperature for 5 min. The mixture was vortexed after a quick addition of 1 mL of concentrated sulfuric acid. After 30 min, the absorbance of the mixture was determined at 490 nm. Glucose at different concentrations of 0.1, 0.2, 0.4, 0.6, 0.8, 1.0 and 1.2 mg/mL were measured with the same protocol. The linear regression equation: Y = 2.8886 x + 0.2022, R2 = 0.9991. x represents the glucuronic acid content and Y represents the sample absorbance.

### 2.4. Uronic Acid Content

The content of uronic acid was determined by the colorimetric *m*-hydroxyl diphenyl protocol [22]. Galacturonic acid aqueous solution with various concentrations (0.1, 0.2, 0.3, 0.4, 0.5, 0.6, 0.8, and 1.0 mg/mL) were prepared to make standard curve. To obtain the color agent, 0.15 g of m-hydroxyl diphenyl was dissolved in 100 mL of 0.5% NaOH solution. To make an acid solution, 0.0125 M borax was added into 98% (*w*/*w*) concentrated sulfuric acid. A 0.4 mL aliquot of each of the polysaccharide samples (1 mg/mL) were distilled in 2.5 mL of the acid solution, then the color agent was added and incubated for 10 min. The absorbance at 520 nm was recorded. The linear regression equation: Y = 0.0003x + 0.0795, R2 = 0.9963. x represents the uronic acid content and Y represents the sample absorbance. All the colorimetric experiments were performed in triplicate.

### 2.5. Molecular Weight Determination

The molecular weight of APS was determined by high-performance gel permeation chromatography according to the protocol of Afiya John et al. [23]. A Shimadzu LC-20 A liquid chromatograph (Shimadzu Corporation, Kyoto, Japan) was used in combination with three tandemly linked G6000PWXL, G5000PWXL, and G3000PWXL columns (Tosoh Bioscience, Stuttgart, Germany). A refractive index detector was used for monitoring. The column was eluted with 20 mM phosphate buffer (pH 7.0) at a flow rate of 0.5 mL/min. Dextran standards with different molecular weights (5220, 11,600, 48,600, 147,600, 409,800, 667,800, 2,990,000 Da) were applied for making calibration curve. The molecular weight was calculated by using retention time (min).

### 2.6. Analyses of Monosaccharide Composition

Monosaccharide composition was analyzed as described by Liu and Yang [24] with some modifications. Ten milligrams of APS were hydrolyzed by 90% (*w*/*w*) formic acid (5 mL) and 2 M trifluoroacetic acid (5 mL) for 2 h at 100 °C. The remaining acid was removed by flushing with nitrogen and evaporated under reduced pressure. Hydroxylamine hydrochloride (10 mg) and pyridine (2 mL) were added and incubated for 30 min at 90 °C. Then 2 mL of acetic anhydride were added. After incubation for 30 min, distilled water (2 mL) was used to terminate the reaction. The acetylated products were extracted by chloroform (3 mL) three times and distilled water (6 mL) was added to remove the water-soluble impurities. The chloroform extract was analyzed by gas chromatography. An HP-5 capillary column(30 m × 0.32 mm × 0.25 μm) was used for chemical separation and a flame ionization detector was equipped. The column temperature was programmed as follows: holding for 0.5 min at 100 °C, increasing to 140 °C at 20 °C/min, keeping for 5 min at 140 °C, increasing to 160 °C at 10 °C/min, then increasing to 250 °C at 10 °C/min and finally holding for 5 min. Nitrogen was used as the carrier gas.

### 2.7. NMR Spectroscopy Analysis 

APS (30 mg) was dissolved in 0.5 mL of D_2_O and 2 μL of acetone was added for calibration [25]. Bruker DRX-500 spectrometer (Bruker, Rheinstetten, Germany) was used. The following spectra were measured: ^1^H, ^13^C, heteronuclear single quantum coherence spectroscopy (HSQC), heteronuclear multiple bond correlation spectroscopy (HMBC), heteronuclear single quantum coherence-total correlation spectroscopy (HSQC-TOCSY), and H/H correlation spectroscopy (COSY). The chemical shifts were expressed in ppm. The calibration chemical shifts of acetone were set to be ^1^H 2.22 ppm and ^13^C 30.89 ppm. 

### 2.8. Endotoxin Content Analyses

Limulus reagent (0.5 EU/mL) was purchased from Xiamen Bioendo Technology Co., Ltd. (Xiamen, China). The endotoxin test was carried out by the guidelines of the Limulus reagent kit. The sample solution (0.1 mL) was added into 0.2 mL of Limulus reagent. After incubation at 37 °C in the dark for 10 min, 100 µL of chromogenic substrates were added into each tube and incubated for 6 min. Then the azo reagent was added and the absorbance of 545 nm was recorded. The standard curve was plotted according to the manufacturer’s protocol (0.01–0.1 EU/mL). The linear regression equation: Y = 0.1273x + 1.9217, R2 = 0.9977. x represents the endotoxin content and Y represents the sample absorbance.

### 2.9. Determination of DPPH Radical Scavenging Activity

The 2,2-diphenyl-1-picrylhydrazyl (DPPH) radical scavenging activity of APS was evaluated by using the method described previously [26] with some modifications. The DPPH in methanol (100 μM) was prepared freshly, and ascorbic acid was prepared as the positive control. Into 20 µL sample solutions, 180 µL of DPPH in methanol were added. After incubation for 30 min in the dark at room temperature, the absorbance was measured at 517 nm. The vehicle control contained 20 µL of 80% methanol and 180 µL of DPPH solution. The scavenging activity of DPPH radicals by the sample was calculated according to the following equation: DPPH radical scavenging activity (%) = (1 − absorbance of sample/absorbance of control) × 100, and the IC_50_ value was calculated on the scavenging activity against DPPH radicals and expressed as µmol of ascorbic acid per gram of ASP of a sour orange fruit (µmol ascorbic acid/g ASP).

### 2.10. Determination of Oxygen Radical Absorbance Capacity (ORAC)

The ORAC assay was conducted using an adaptation of the method published previously [26]. All the reagents and samples were made freshly and dissolved in 75 mM NaH_2_ PO_4_-Na_2_HPO_4_ buffer (pH 7.4), respectively. The Trolox solutions (6.25, 12.5, 25, and 50 μM) were used as the positive control, and 75 mM phosphate (pH 7.4) was used as the blank. Then, 20 µL of sample solutions were added to the black-walled 96-well microplates (Corning Scientific, Corning, NY, USA) and incubated for 30 min at 37 °C. Afterward, 200 µL of fluorescein sodium salt (0.96 µM) and 20 µL of ABAP (119 mM) were added. The reaction was measured immediately at 37 °C. The fluorescence generation was monitored every 1.5 min for 60 cycles by using a multi-mode microplate reader (excitation wavelength 480 nm, and emission wavelength 520 nm). The ORAC values were expressed as µmol of Trolox equivalents per gram of ASP of sour orange fruit (µmol Trolox equiv./g ASP) on the basis of the regression equation between Trolox concentration and the net area under the curve (AUC).

### 2.11. Statistical Analyses

All the experiments were performed in triplicate and the results were expressed as mean ± standard deviation. Significant differences were tested by one-way analysis of variance. Statistical differences with *p* < 0.05 were considered to be significant. All experiments were performed in triplicate. The results were expressed as arithmetic means ± standard deviation (SD) of each independent experiment performed in triplicate.

## 3. Results and Discussion

### 3.1. Appearance, Content, and Monosaccharide Composition 

**Figure 1:**
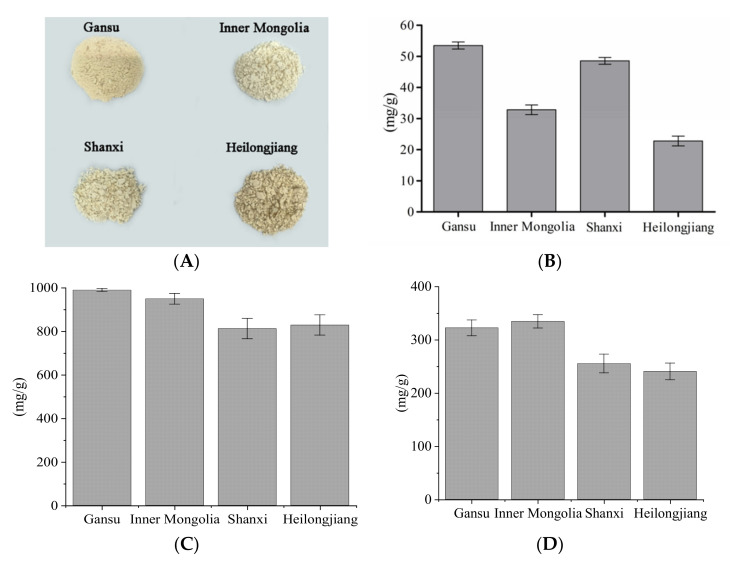
The appearance and yields of *A. membranaceus* polysaccharides from four origins (Gansu, Inner Mongolia, Shanxi, and Heilongjiang). (**A**), appearance; (**B**), polysaccharide yield; (**C**), polysaccharide content; (**D**), uronic acid content.

**Figure 2:**
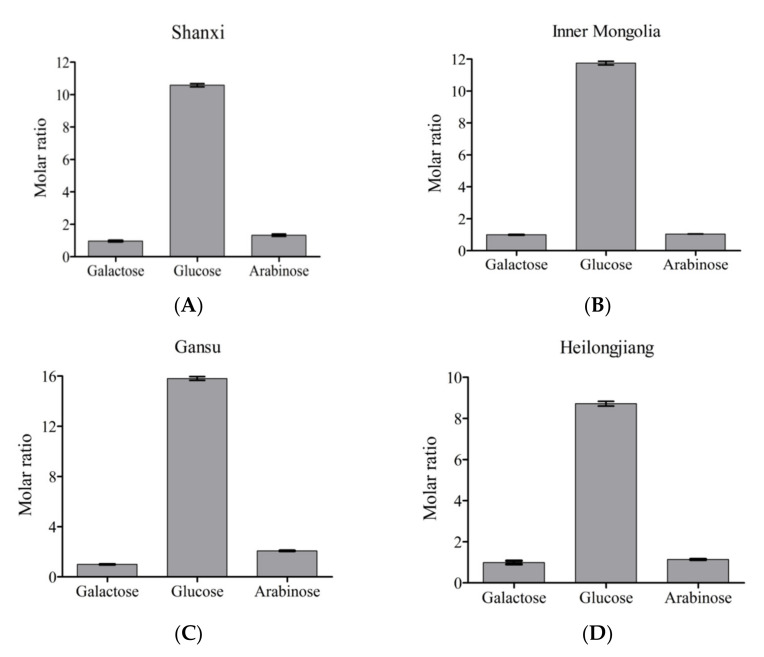
Monosaccharide compositions of APSs from four origins. (**A**), Shanxi; (**B**), Inner Mongolia; (**C**), Gansu; (**D**), Heilongjiang.

The appearance and yields of APSs isolated from four origins are shown in Figure 1. The color of polysaccharides from four origins (Gansu, Heilongjiang, Inner Mongolia, Shanxi) was detected by a colorimeter, and the values of L*, a*, and b* are shown in Table 1. Three readings of “L” (lightness), “a” (redness), and “b” (yellowness) indicate the lightness coefficient, red color coefficient, and yellow color coefficient, respectively. Differences values of L*, a*, and b* were used to evaluate the change in different color parameters. The results indicated that ‘Heilongjiang’ polysaccharide extract had a more yellow-green color (b*) than the other origins sample. The yield of APS was determined. It was in decreasing order, ‘Gansu’ > ‘Shanxi’ > ‘Inner Mongolia’ > ‘Heilongjiang’. The ‘Gansu’ polysaccharide extract had the highest content of polysaccharides (Figure 1C). In Figure 2, the neutral monosaccharide compositions were determined by gas chromatography. Compared with the monosaccharide standard, APS from different origins had identical monosaccharide compositions, including arabinose, glucose, and galactose. By combining the NMR data, galacturonic acid was found as the only uronic acid in APS. The content of galacturonic acid was detected by the colorimetric m-hydroxyl diphenyl protocol and the results showed that ‘Gansu’ and ‘Inner Mongolia’ had higher uronic acid levels. Furthermore, glucose was the major monosaccharide due to the high molar ratio in all samples. It is worth mentioning that the water solubility of APS was in decreasing order, ‘Shanxi’ > ‘Gansu’ > ‘Heilongjiang’ > ‘Inner Mongolia’. The endotoxin (liposaccharide) was determined in this work. All the samples had endotoxin levels below 0.1 EU/mL. It indicated that these polysaccharides were in the safe range.

The properties of medicinal plants from different origins varied due to the environmental effects [27]. It can influence their appearance, composition, and bioactivities. ASP from four origins presented significantly different colors according to chrominance index (L*, a*, and b* in Table 1). It was correlated with their quality and purity. Different geographic locations, edaphic traits, and climatic conditions were critical factors contributing to the content and structure characteristics of polysaccharides [28]. It has been reported that the polysaccharides in *A. membranaceus* from Gansu present the highest yield compared to the other places [6]. This is consistent with the results of this work (Figure 1B). Four monosaccharides were detected in APS, including arabinose, glucose, galactose, and galacturonic acid (Figure 2). Li et al. [29] have reported the same monosaccharide composition. However, the molar ratios of the four monosaccharides were different. Arabinose, glucose, galactose, and galacturonic acid were common monosaccharides presented in plant-derived polysaccharides [30], such as the *Ziziphus jujuba, Glycyrrhiza,* and *Ginkgo* polysaccharides [31]. They tend to present the antioxidant and immunoregulation activities by providing a protective effect against oxidative stress caused by H_2_O_2_ and regulation through Toll-like receptor 4 (TLR4) and its downstream p38 signal pathway [32]. As determined in this work, glucose was the leading monosaccharide in APS (Figure 2). It is the unique unit of starch and cellulose that widely occurs in grains and crops [33]. Furthermore, it is the component of bioactive polysaccharides, including β-1,3-glucan, α-1,6-glucan, and heteroglucans [34]. Galacturonic acid constructs the backbone of pectins [35], a bioactive macromolecule, which is presented in all the plant cell walls. Uronic acid residues have been well established to alter physicochemical properties and solubility of polysaccharides and play an important role in polysaccharides functions such as anti-complement activity [36]. Arabinose and galactose can also be the components of pectins, and other bioactive polysaccharides, like arabinogalactans [37]. To understand which polysaccharides are in APS, NMR analysis is required.

### 3.2. Molecular Weight Profile of APS

**Figure 3:**
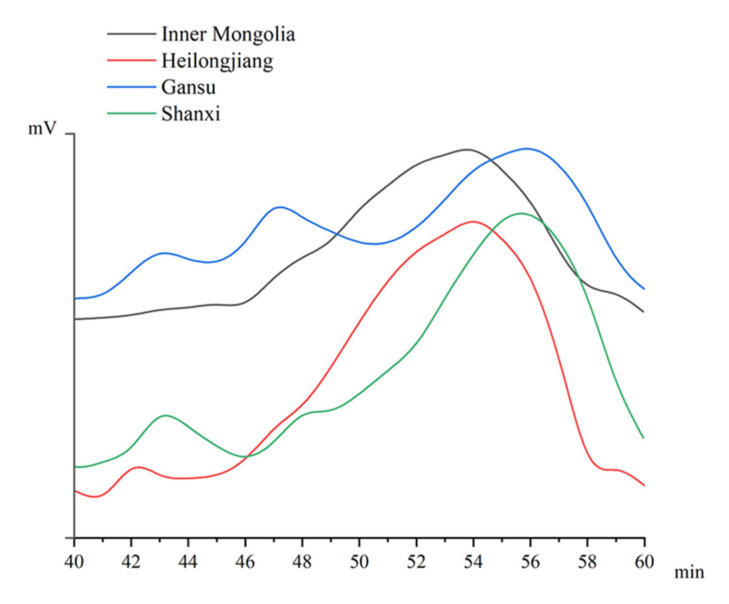
The molecular weight profiles of APSs from four origins (Gansu, Heilongjiang, Inner Mongolia, and Shanxi).

High-performance gel permeation chromatography was applied to determine the molecular weight profile of APS. As shown in Table 2 and Figure 3, APS could be divided into three fractions based on retention time or molecular weight. The first fraction was eluted between 40–45 min, with the molecular weight in the range of 700–1200 kDa. The second fraction was eluted between 46–50 min, with the molecular weight in the range of 160–300 kDa. The third fraction was eluted between 52–57 min, with the molecular weight in the range of 20–40 kDa. The molecular weight profiles of four APSs were apparently different. The first fraction of APS from Shanxi showed the largest molecular weight, which was 1152 kDa, while that of Inner Mongolia had the smallest molecular weight of 746 kDa. Moreover, by area integration, the proportion of this fraction from Shanxi was higher than those from Heilongjiang and Inner Mongolia. For each APS, the third fraction accounted for the largest proportion. It indicated that this was the dominant polysaccharide fraction. The proportions of four APSs were in the range of 62–80%. It is worth mentioning APS from Inner Mongolia, which was the most famous origin of *A. membranaceus* in China. The first and second fractions were not obvious in the gel permeation chromatogram. The proportion sum of the second and third fractions was over 97%. From the viewpoint of molecular weight distribution, the most similar composition to Inner Mongolia APS was that from Heilongjiang.

Molecular weight is an important structural feature of a polysaccharide. It is highly associated with biological activity and function [15]. Xu et al. obtained an ASP with molecular weight in the range of 100–120 kDa [38], while the fraction with molecular weight exceeded 500 kDa was reported in another research [29]. The origin and extraction technique might be responsible for these results. Previous research has reported that the polysaccharides with a low molecular weight below 50 kDa usually present better antioxidant and immunomodulatory activities than those with large molecular weight [39]. Ren et al. have elaborated that the number-average and weight-average molecular weight of *Astragalus* polysaccharides significantly decreased when treated by gamma irradiation, and the lower molecular weight ASP exhibited the highest ability on immunomodulatory properties and could up-regulate the expression of inflammatory cytokines [40]. It might be due to easy absorption and utilization by microbes in the intestinal tract [41]. As determined in this work, the dominant polysaccharide fraction of APS had a small molecular weight of 20–40 kDa (Table 2). The abundance of small molecular weight polysaccharides might contribute to the great health benefits of *A. membranaceus*.

### 3.3. Structural Characteristics Identified by NMR

**Figure 4:**
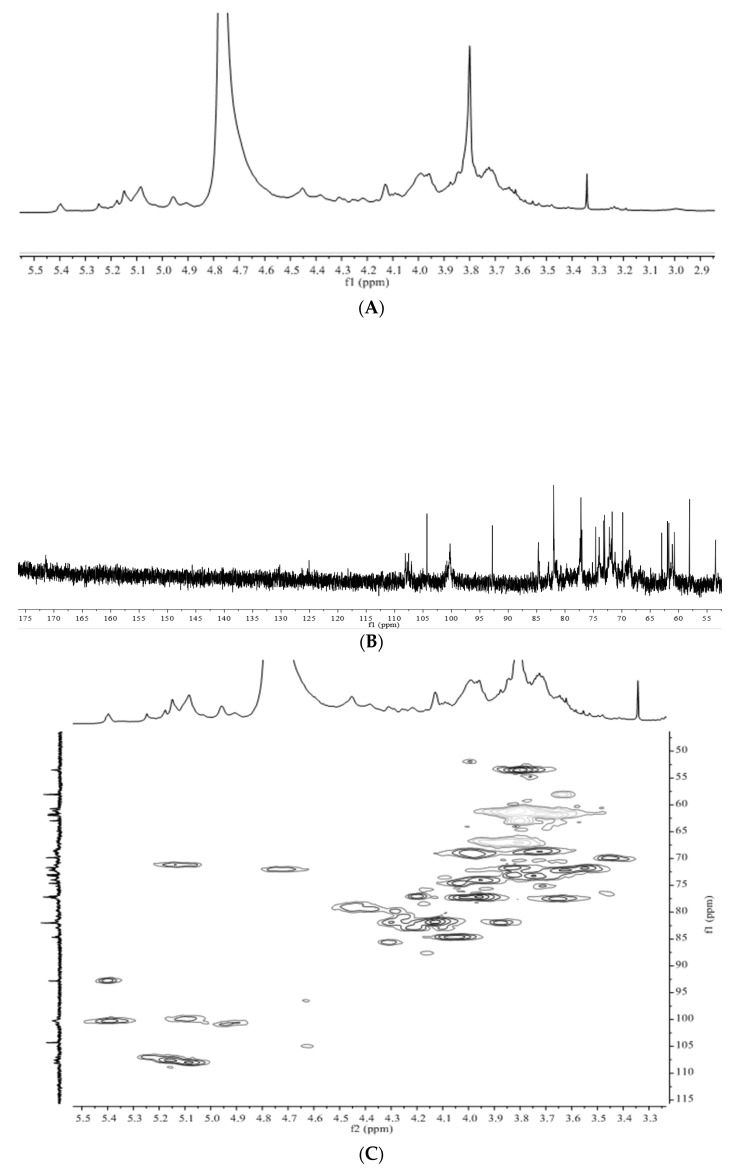

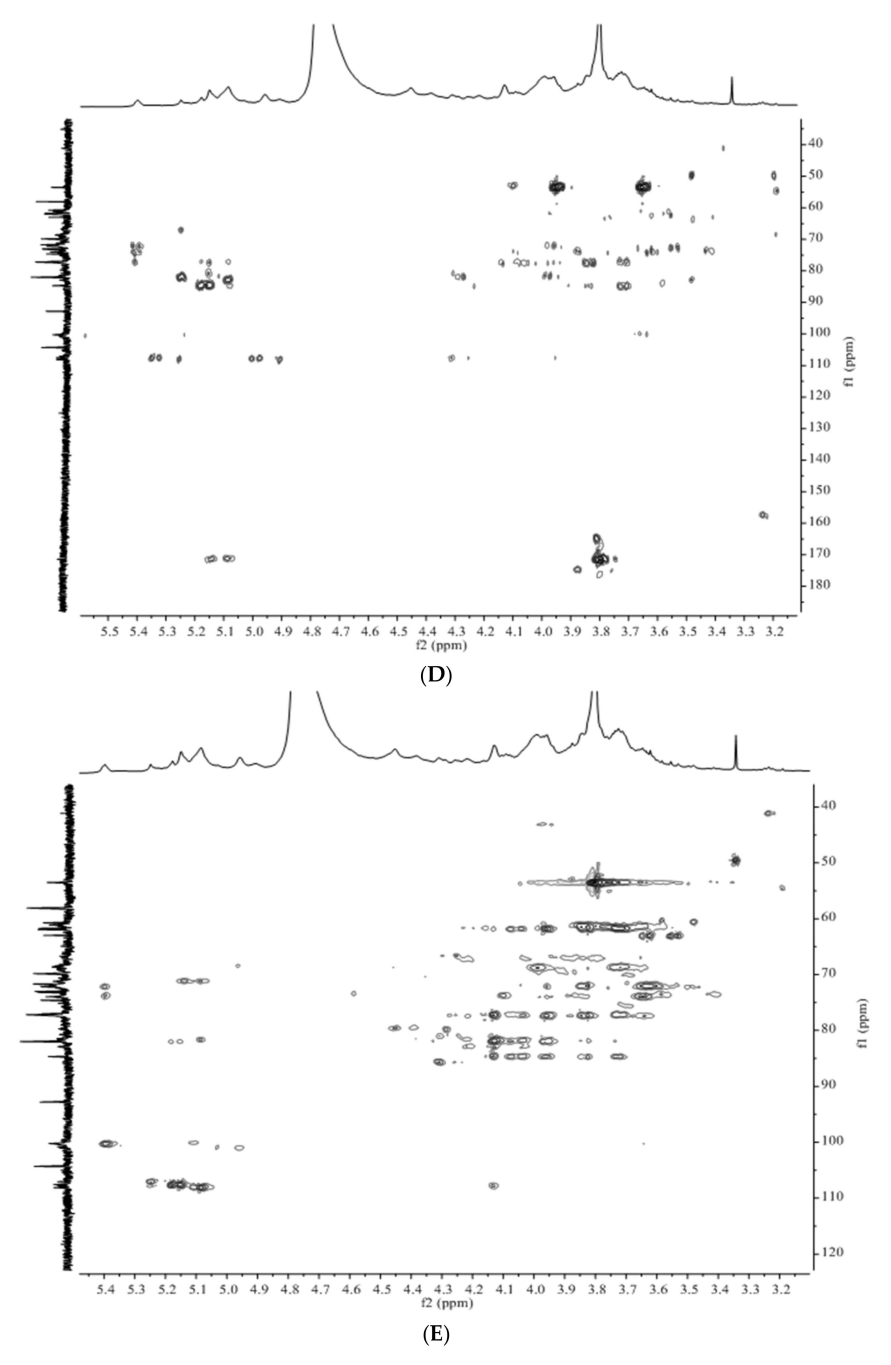

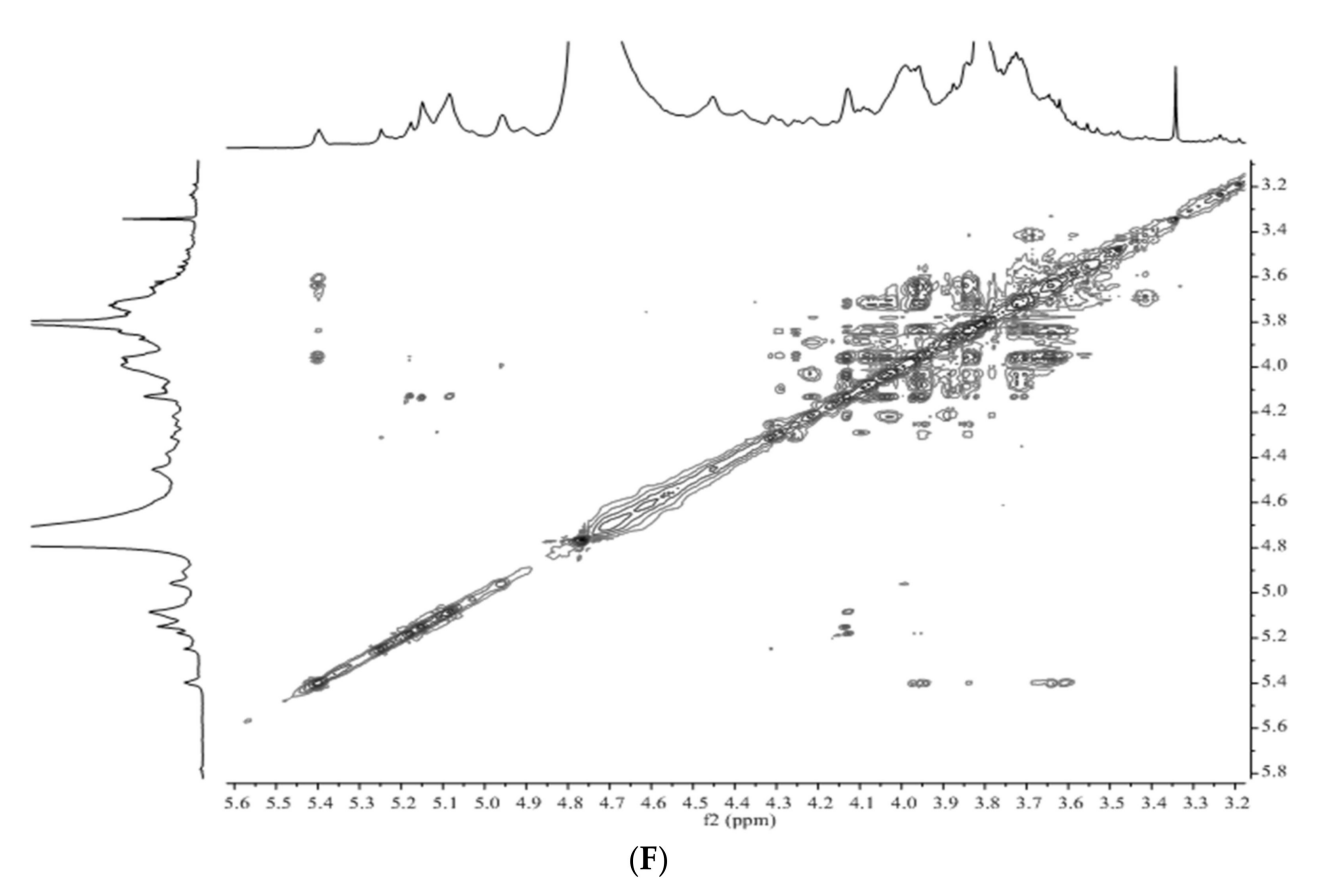
NMR spectra of APS. (**A**), 1H spectra; (**B**), 13C spectra; (**C**), HSQC spectra; (**D**), HMBC spectra; (**E**), HSQC-TOCSY spectra; (**F**), COSY spectra.

The structural characteristics of APS were identified by 1D and 2D NMR spectra, including ^1^H, ^13^C, HMBC, COSY, HSQC, and HSQC-TOCSY spectra. The spectra are shown in Figure 4. Seven glycosidic residues were designated by assigning anomeric signals (H/C) at 5.39/100.3, 5.25/106.9, 5.15/107.6, 5.09/108.0, 5.10/99.9, 4.94/100.9, and 4.62/104.9. The H/C peak at 5.39/100.3 was the characteristic anomeric signal of →4)-α-Glc*p*-(1→, the unique glycosidic linkage in α-1,4-glucan, which could be starch or dextrin. H-2 had a chemical shift of 3.61 by checking COSY spectra. HSQC-TOCSY spectra further revealed the chemical shifts of C-2 and C-3, which were 72.1 and 73.8, respectively. These data were further confirmed by HMBC spectra. The cross peak of H-1/C-4 was presented in HMBC spectra at 5.39/77.7. This could be an important and direct evidence of α-1,4-glucan. The H-5/C-5 (3.84/72.0) and H-6/C-6 (3.80, 3.88/60.7) were assigned by resolving several 2D spectra. There were two anomeric signals, 5.40/92.8 and 104.3, in HSQC spectra, which were distant from other anomeric signals. They were the characteristic signals for sucrose [42]. The former was from α-Glc*p*-(1→ and the latter was from C-2 of β-Fru*p*. As Fru*p* was easily degraded during acid hydrolysis, it was not detectable when analyzing monosaccharide composition.

Ara*f* is an important monosaccharide presented in pectins and arabinogalactan proteins. The chemical shift of anomeric carbon is usually close to 110. This characteristic signal makes Ara*f* easy to identify. Three anomeric signals at 5.25/106.9, 5.15/107.6, 5.09/108.0, were assigned to α-Ara*f*. For an anomeric signal at 5.09/108.0, H-2 was 4.13 by checking COSY spectra. C-2 was 81.7 by checking HSQC-TOCSY spectra. HMBC spectra showed that C-3 was 77.3. These data indicated that C-2 and C-3 were not involved in glycosidic linkage by comparing with free α-Ara*f*. Another strong cross peak at 5.09/82.9 in HMBC spectra was assigned to H-1/C-4. The upfield shift of C-4 indicated that C-5 was involved in glycosidic linkage. The HSQC-TOCSY spectra further confirmed H-5a,5b/C-5 to be 3.80,3.88/66.9. Therefore, this glycosidic linkage was identified to be →5)-α-Ara*f*-(1→.

For the anomeric signal at 5.15/107.6, HMBC spectra showed two cross-peaks at 5.15/84.8 and 5.15/77.6, respectively, which were assigned to H-1/C-4 and H-1/C-3. Further checking HSQC-TOCSY spectra revealed the chemical shifts of H-5a,5b/C-5 were 3.72,3.80/61.9. This information indicated that this glycosidic linkage was α-Ara*f*-(1→. Another cross peak at 5.15/80.5 indicated that this was linked to another sugar unit and 80.5 might be the chemical shift of C2-C4 from Gal*p*A or Gal*p*.

For the anomeric signal at 5.25/106.9, HMBC spectra revealed two cross-peaks at 5.25/67.3 and 5.25/81.7, respectively. The former signal indicated that H-1 of α-Ara*f* was linked to C-5 of another α-Ara*f*. The second cross peak indicated that C-2 of α-Ara*f* had a chemical shift of 81.7. By comparing with free α-Ara*f*, it could be deduced that C-3 was not involved in glycosidic linkage. This information suggests that this sugar presented as α-Ara*f*-(1→ or →5)-α-Ara*f*-(1→.

Gal*p*A was presented as a leading signal in HSQC spectra. The C-6 chemical shifts at 171.6 were characteristic for the carboxyl group of Gal*p*A. The value of 171.6 indicated that this carboxyl acid was methylated to form methyl ester. The cross peak in HMBC spectra at 3.80/171.4 further confirmed the presence of methyl ester. The methyl signal at 3.80/53.6 was clear in HSQC spectra. The signal of unsubstituted carboxylic acid near 175 was very weak in ^13^C and HMBC spectra. It indicated that most of Gal*p*A residues were presented as methyl ester. Two characteristic anomeric signals (5.09/100.1 and 4.96/100.8) were assigned to →4)-α-D-Gal*p*A-(1→ and →4)-α-D-Gal*p*AMe-(1→, respectively. For →4)-α-D-Gal*p*AMe-(1→, the other H/C chemical shifts were 3.73/68.7, 3.96/69.1, 4.44/79.3 and 5.14/71.2. For →4)-α-D-Gal*p*A-(1→, the other H/C chemical shifts were 3.74/69.2, 3.99/70.0, 4.41/79.3 and 4.74/72.1. Both glycosidic linkages constructed the α-(1→4)-galacturonan backbone of pectins. 

A unique anomeric signal at 4.63/105.0 was observed in HSQC spectra, which might be assigned to β-Gal*p* or β-Glc*p*. The H-2 had a chemical shift of 3.76 when observing COSY spectra. C-2 was further identified to be 70.6 by checking HSQC spectra. These data indicated that this sugar should be β-Gal*p*. As this sugar unit had a weak signal in 2D spectra, the glycosidic linkage could not be identified.

As mentioned above, Gal*p*A, Ara*f* and Gal*p* constituted the main polysaccharides in *A. membranaceus*, in which Gal*p*A constructed the backbone and Ara*f* constructed the side chains. Pectins usually have three types of structure [43]. Homogalacturonan is a linear polysaccharide consisting of α-(1→4)-D-galacturonan. Rhamnogalacturonan I consists of →4)-α-D-Gal*p*A-(1→2)-α-L-Rha*p*-(1→ backbone with side chains principally comprised of Ara*f* and/or Gal*p* linking to C-4 of Rha*p*. Rhamnogalacturonan II has a backbone of homogalacturonan with complexed side chains linking to Gal*p*A. As no Rha*p* was detected in APS and Gal*p*A/Ara*f*/Gal*p* were the main monosaccharides, rhamnogalacturonan II was identified as the pectin in APS. Only one glycosidic linkage was detected for Glc*p* and it constructed a homoglucan, α-(1→4)-glucan. This information suggested that the water-soluble polysaccharides in *A. membranaceus* were rhamnogalacturonan II and α-(1→4)-glucan.

### 3.4. Structure Differences of APS from Four Origins 

**Figure 5:**
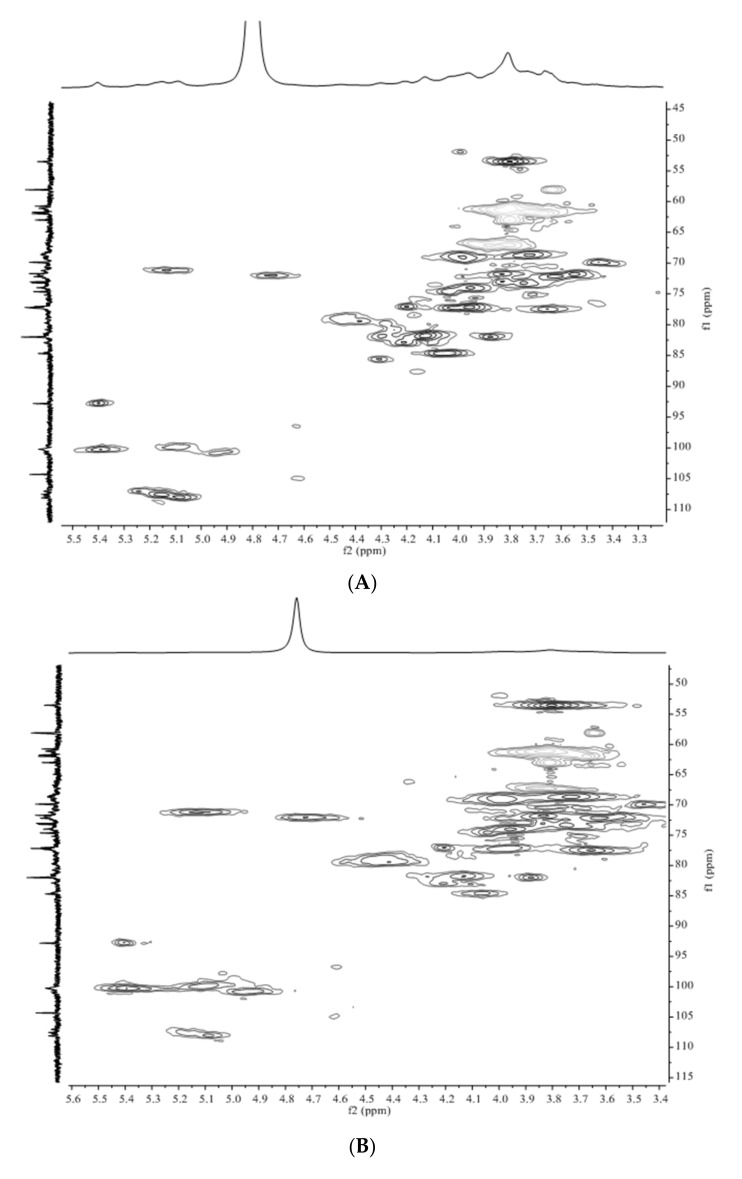

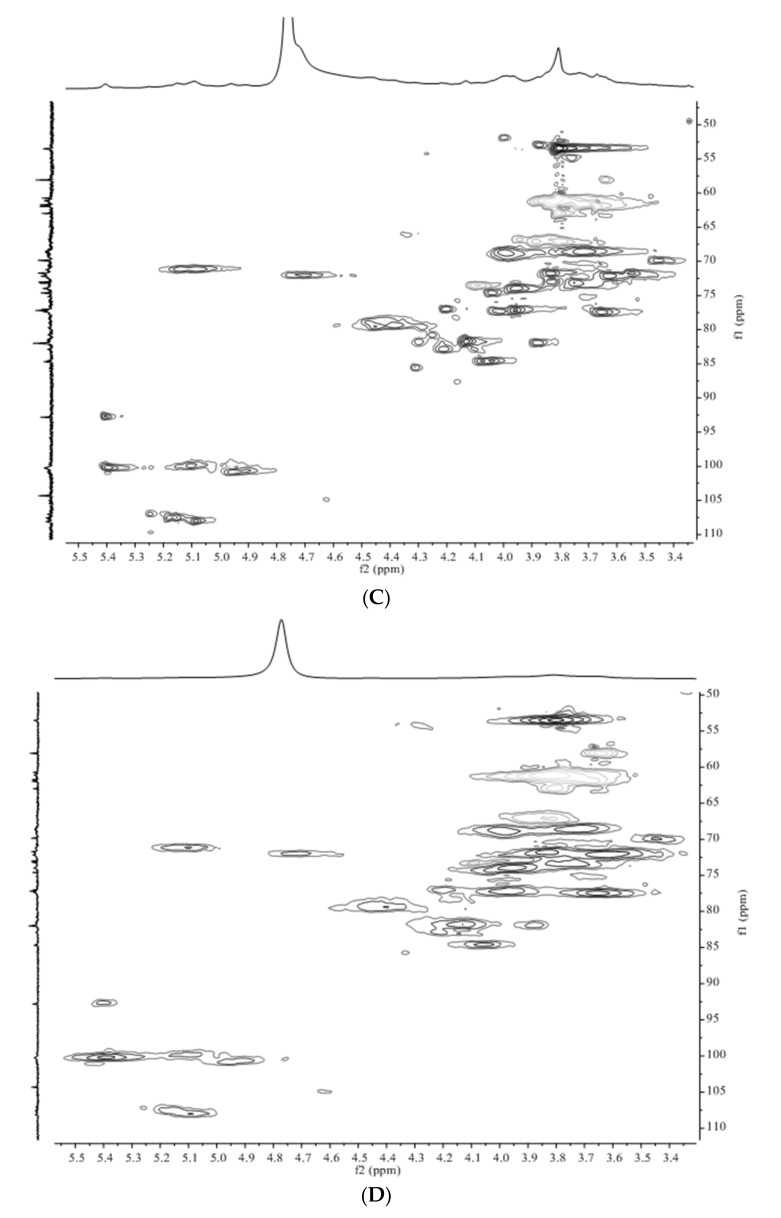

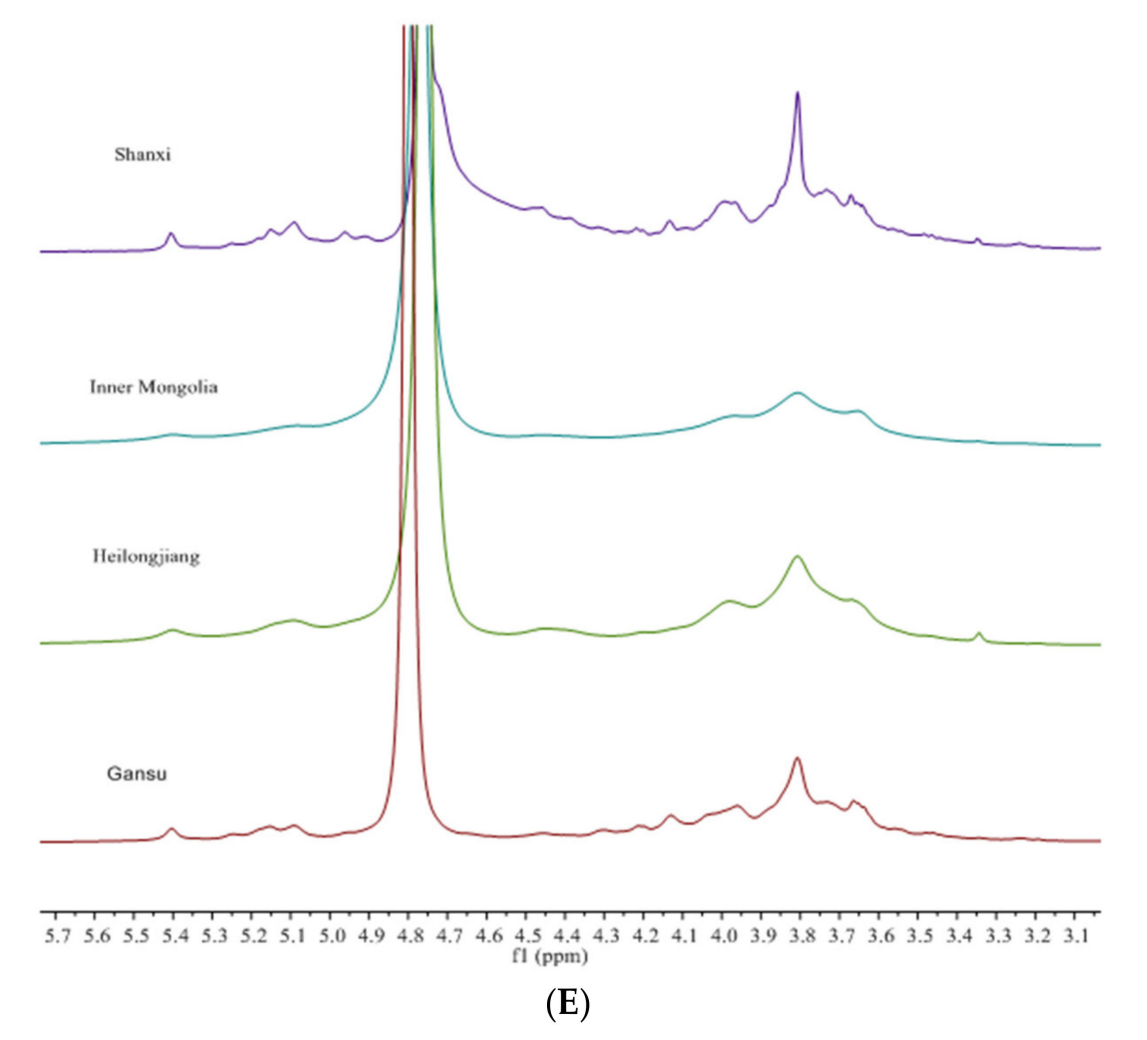
NMR spectra of APS. (**A**), HSQC spectra of APS from Gansu; (**B**), HSQC spectra of APS from Heilongjiang; (**C**), HSQC spectra of APS from Shanxi; (**D**), HSQC spectra of APS from Inner Mongolia; (**E**), ^1^H spectra of four APS samples.

To reveal the structural differences of APS from different origins, HSQC spectra of four APS samples were recorded to reflect the structure information. The HSQC spectra of four APS samples are shown in Figure 5. However, there were no significant differences among the four HSQC spectra. The predominant anomeric signals of α-Ara*f*-(1→, →5)-α-Ara*f*-(1→, →4)-α-Gal*p*A-(1→, →4)-α-Gal*p*AMe-(1→ and →4)-α-Glc*p*-(1→ were all detected in four APS samples. These results suggested that the basic structural characteristics of *A. membranaceus* polysaccharides remained consistently when growing in different origins. 

It was worthy to record the intensity of methyl ester in ^1^H spectra (Figure 5E). The chemical shifts of methyl ester were 3.80/53.6 (H/C). By comparing the intensity of ^1^H at 3.80, they varied among the four APS samples. The APS from Shanxi showed the highest intensity, followed by those from Heilongjiang and Gansu. Inner Mongolia APS had the lowest content of methyl ester. The level of methyl ester in APS was highly related to solubility in water. This explained the highest water solubility of Shanxi APS and the lowest water solubility of Inner Mongolia APS when comparing four APS samples. 

It has been documented that polysaccharides can be absorbed by the small intestine and colon [44]. They can bind to specific receptors, activate the downstream pathway, and exert bioactivities [45]. Pectins can inhibit cancer progression by inhibiting Gal3 (a unique member of a family of evolutionary conserved galactose-binding lectins) [46]. The interaction with colon bacteria is another mechanism of pectins to show immunomodulatory activity [47]. Solubility is an important physical property of bioactive polysaccharides. It can influence the absorption and metabolism of bioactive polysaccharides in vivo. The presence of methyl ester and its level has a certain effect on the health benefits of pectins by modifying the metabolism in vivo.

### 3.5. Antioxidant Activity 

DPPH is a stable nitrogen-centered free radical based on a single electron transfer reaction. ORAC stands for oxygen radical absorbance capacity based on hydrogen atom transfer reaction [48]. It has been widely used to evaluate the antioxidant activity of polysaccharides. In this study, the ORAC assay and DPPH radical scavenging activity of APS were tested, and the antioxidant values are shown in Table 3. Using the ORAC assay for hydrophilic compounds, the order of activity was ‘Gansu’ > ‘Inner Mongolia’ > ‘Shanxi’ > ‘Heilongjiang’, while the DPPH radical scavenging activity was in a decreasing order, ‘Heilongjiang’ > ‘Gansu’ > ‘Shanxi’ > ‘Inner Mongolia’. It confirmed that APS show obvious antioxidant activity. 

The antioxidant activity of polysaccharides is usually associated with monosaccharide composition, the content of uronic acid, and molecular weight [11]. Different origins of astragalus samples presented different antioxidant capacities on ORAC and DPPH (Table 3). *Laminaria japonica* polysaccharides with higher uronic acid content presented better ORAC activity [49]. Moreover, it should be mentioned that the low level of phenolics presented in the polysaccharide extract should have a contribution to the antioxidant activity detected. 

## 4. Conclusions

As mentioned above, the yields of APSs from four origins were significantly different. However, their monosaccharide compositions were identical, including Galp, Glcp, Araf, and GalpA. By analyzing 1D and 2D NMR data, the polysaccharide compositions of APSs from four origins and their glycosidic linkages were also identical. Rhamnogalacturonan II pectins and α-(1→4)-glucan were the dominant polysaccharides. However, the level of methyl ester varied to a large extent among four APS samples. The molecular weight profile was also different, and Inner Mongolia APS had the largest percentage of small molecular-weight polysaccharides. These data indicated that molecular weight and level of methyl ester were two important parameters determining the difference of APSs from four origins, which might further influence the health benefits of *A. membranaceus*. In future work, it is worth investigating the cellular bioactivities of APSs from four origins and the mechanisms underlying them.

## Figures and Tables

**Table 1:** Chrominance of APS fractions.

Origins	Chrominance			
	L*	a*	b*	ΔE
Inner Mongolia	78.65 ± 2.46 ^a^	1.53 ± 0.10 ^a^	6.42 ± 0.34 ^a^	0 ^a^
Shanxi	80.51 ± 0.28 ^a^	1.79 ± 0.03 ^b^	8.35 ± 0.36 ^b^	2.69 ± 0.44 ^a^
Gansu	73.51 ± 2.03 ^b^	2.27 ± 0.04 ^c^	9.00 ± 0.87 ^b^	5.80 ± 1.97 ^b^
Heilongjiang	70.48 ± 3.92 ^b^	2.08 ± 0.02 ^d^	13.11 ± 0.45 ^c^	10.57 ± 2.61 ^c^

Values with no letters in common are significantly different (*p* < 0.05).

**Table 2:** Percentages of APS fractions with different molecular weights.

Origins	Molecular Weight (kDa)		
	20–40	160–300	700–1200
Gansu	62.9%	23.0%	14.1%
Heilongjiang	76.6%	19.4%	4.0%
Inner Mongolia	75.7%	22.1%	2.2%
Shanxi	80.4%	7.4%	12.2%

**Table 3:** Antioxidant activity of APS from four origins.

Origins	ORAC Values (μmol Trolox Equiv/g ASP)	IC50 Values Against DPPH (μmol Ascorbic Acid/g ASP)
Inner Mongolia	192.03 ± 6.12 ^a^	1.48 ± 0.10 ^a^
Shanxi	43.66 ± 3.32 ^b^	2.82 ± 0.08 ^b^
Gansu	201.91 ± 8.33 ^a^	3.38 ± 0.12 ^c^
Heilongjiang	17.91 ± 2.09 ^c^	3.43 ± 0.06 ^c^

Values with no letters in common are significantly different (*p* < 0.05).

## Data Availability

All data presented are available in the manuscript.
